# “The Stakes Are Higher”- Patient and Caregiver Perspectives on Cystic Fibrosis Research and Personalized Medicine

**DOI:** 10.3389/fmed.2022.841887

**Published:** 2022-03-23

**Authors:** Terese Knoppers, Marie Cosquer, Julie Hagan, Minh Thu Nguyen, Bartha Maria Knoppers

**Affiliations:** ^1^Centre of Genomics and Policy, McGill University, Montreal, QC, Canada; ^2^Department of Sociology, Laval University, Quebec, QC, Canada

**Keywords:** patient perspectives, caregiver perspectives, cystic fibrosis, personalized medicine, ethics, translation, access, qualitative

## Abstract

**Introduction:**

Making bench to bedside advances in cystic fibrosis (CF) care requires the sustained engagement and trust of people living with CF. However, there is a scarcity of studies exploring their concerns and priorities regarding research and its end products. The aim of this qualitative study was to generate empirical evidence regarding patient and caregiver perspectives on cystic fibrosis research and personalized medicine to foster developments in translational research in Canada.

**Methods:**

A total of 15 focus groups were conducted, engaging 22 adults with CF and 18 caregivers (e.g., parents, siblings and partners) living in Canada. Inductive thematic analysis relied on an iterative process involving themes derived from both participant meaning-making and existing scientific literature. Participant perspectives were considered along intrapersonal, intracommunity, interpersonal, and structural lines.

**Results:**

Overall, participants described a relationship to CF research inextricable from the lived experience of CF as a lifelong progressive and terminal disease and from the goal of advancing medical science. They were enthusiastic and excited about the emergence of CFTR modulators, although they had some knowledge gaps regarding the associated research. They largely spoke to positive experiences with researcher communication but had feedback regarding informed consent processes and the return of study results. Participants also voiced concerns about structural access barriers to research and to its end products. Extensive histories of research participation, a relatively small and intercommunicative CF community, and structural overlap between research and care settings contributed to their perspectives and priorities.

**Conclusion:**

Study findings are valuable for researchers and policy-makers in CF and rare or progressive diseases more broadly. Continuing to solicit and listen to the voices of patients and caregivers is crucial for research ethics and the translation of new therapies in the area of personalized medicine.

## Introduction

*The reasons for participating [in research] are number one I am a big believer in public science and public information. I really believe that research is crucial in informing medical decisions. But I think that the stakes are higher when you’re dealing with a rare disease in which there are fewer patients, and in a disease like this that is progressive, there’s also some urgency to figuring out as quickly as possible ways that we can improve bench to bedside protocols and care* (C17).

Cystic fibrosis (CF) is a degenerative multi-system disease caused by pathogenic variants in the cystic fibrosis transmembrane conductance regulator (*CFTR*) gene (1). In Canada, there are approximately 4,300 people living with CF, and CF occurs in an estimated 1 out of 3,600 live births (2). CF causes digestive disorders and progressive lung disease due to recurrent and chronic lung infections, resulting in a shortened lifespan. There are also significant psychosocial and economic impacts for people living with CF, their caregivers, and their families, associated with the intensive ongoing need for medical care and CF’s episodic yet progressive symptomology (3).

Advances in diagnosis, therapies, and multi-disciplinary healthcare have greatly improved the quality of life and life expectancy for people with CF over the past decades (3, 4). Treatment focuses on slowing lung function deterioration and addressing digestive system problems. Lung transplants often also become necessary for people with advanced CF. Recently, upstream therapies have been developed to overcome the pathogenic variants that cause CF, improving or even restoring the functioning of the *CFTR* gene (5). These CFTR modulators, which offer treatment to individuals with certain variants, represent a paradigm shift in personalized medicine and disease prognosis for patients with CF (5, 6). The latest to be approved by Health Canada, Trikafta, is particularly ground-breaking in that an estimated 90% of Canadians with CF could benefit from it (7).

Making advances in CF care requires the sustained engagement and trust of people living with CF in research, not least because the population is small (and made smaller due to stratification during research) and patients with CF are already intensively studied. There is a scarcity of studies exploring the relationship of the CF community to research: their perspectives, concerns, and priorities [see (8–10)]. Such a study is particularly appropriate as the field branches into the development of personalized medicine (11). This paradigm shift raises the ethical stakes of research with the CF population because of the cost of the medicines being produced (an estimated $300,000 per person per year) and because the stem cell and genomics research producing this personalized medicine remain widely misunderstood domains (12–15). It is therefore critical and timely that we consider the lived experience of people with CF in research and its translation into personalized medicine (16). We conducted a qualitative study in order to generate empirical evidence regarding patient and caregiver perspectives on the utility and limitations of CF research and personalized medicine in Canada. Findings are valuable for researchers and policy-makers not only in the area of CF but in rare and/or progressive diseases more broadly.

## Materials and Methods

### Recruitment and Study Participants

Given that the number of adults living with CF in Canada is small, we used a convenience sampling strategy to select participants for our study. Our main method of recruitment was through CF Canada and the different local chapters of the organization. We posted our recruitment ads via their Facebook pages as well as their members’ email lists. We were also able to recruit through the assistance of several CF clinics across Canada, most notably in Ontario and Quebec. We targeted participants 18 years or older with a CF diagnosis. As most cases of CF are detected very early in life and about 40% of patients with CF are children (2), we decided to seek the opinions and experiences of caregivers (e.g., parents, siblings and partners) in our study to enrich our data. In both cases, we aimed to enroll individuals who had already participated in research and/or contributed to biobanks, databases, or registries in the past, but this was not an exclusion criterion.

Interested participants contacted the Centre of Genomics and Policy via phone and/or email. Those who met the recruitment criteria were sent more information about the study and the study topics as well as the sociodemographic survey and consent form. They were also given an opportunity to ask any initial questions. Focus groups were then organized in sequence of interest by participant type and by language. Recruitment continued until a clear pattern emerged across focus groups and we determined that we had reached data saturation (17).

In total, 22 adults with CF and 18 caregivers participated in our study. Four focus groups took place in French and eleven in English. Participants came from eight Canadian provinces (Alberta, British Columbia, Manitoba, Ontario, New Brunswick, Nova Scotia, Quebec, and Saskatchewan) with half residing in Ontario. Not all participants elected to submit demographic information, so our data is partial. But among our completed socio-demographic surveys most participants were racially White, and a broad range of income levels and educational backgrounds were represented.

### Focus Groups

Between March 2019 and March 2021, we conducted fifteen focus groups with patients and caregivers. Six of these focus groups were composed of patients (including an asynchronous online forum held in March 2019) and seven were composed of caregivers of patients with CF. We held two mixed groups to accommodate participants, for example, when an adult patient and their partner wished to participate in the discussion as a couple. In part because of infection control guidelines to prevent person-to-person transmission of pathogens among people with CF (18) and in part because we were adapting to changing research conditions after the onset of COVID-19, our study used a mix of online and in-person, synchronous and asynchronous focus groups. Most focus groups (13/15) were synchronous and online.

Focus groups were led by JH, MC, and MTN, all of whom had previous experience. They began with a discussion of the project and consent form and ended with time for further questions, comments, and debriefing. SA and SF assisted as note-takers. Focus groups explored the perceptions, expectations and priorities of patients and caregivers concerning CF research. Specifically, we asked about their motivation to participate in different studies, their previous research experience, and their communication preferences around research participation. We also discussed their knowledge and perceptions regarding stem cells and genetic-based research to help individualize drug treatments and the value such research may have for patients and their families. Finally, we solicited their opinions about the risks and benefits of contributing to biobanks and the use of cells and tissues, including issues related to confidentiality.

Six adult patients participated in the asynchronous online focus group, which consisted of a seven-day text-based discussion supported by iTracks, a Canadian software company. Participants were given a series of consecutive questions they could answer at their own leisure. A moderator from our team encouraged dialogue by reacting to participant answers and posting new questions and further prompts. Asynchronous focus groups have been used in health research to reach populations with chronic health conditions as the time flexibility of asynchronous discussion can accommodate fatigue and changes in condition (19, 20). The questions used for the asynchronous discussion forum were the same as those used in the focus group script, with minor adaptations. The synchronous focus groups were semi-structured group conversations held via videoconferencing solutions. They consisted of a 1–4 participants, a moderator, and a note-taker, and lasted two hours on average. In two instances, the focus group exceptionally consisted of only one participant due to last minute withdrawal of other participants. Since the focus groups were conducted online, we found that smaller groups were more likely to encourage interactions. Synchronous focus groups were audio recorded and outsourced to a professional transcription service.

### Analysis

Two members of the researcher team (JH and SA) analyzed the transcriptions of the focus groups using the qualitative content analysis software NVivo (1.5). Thematic analysis relied on an iterative process engaging themes derived from the existing scientific literature (with themes emerging from the data). First, JH and SA developed content areas that reflected the general topics discussed in the focus groups. Then, they developed themes, sub-themes, and codes from the participants’ unit of meaning by coding approximately half of the focus group content. At the end of a process of reflection and discussion, agreed-upon definitions were given to each theme and sub-theme, and a codebook was created. JH then coded the second half of the focus groups. As a final step, TK and MC were able to review the coded data, themes, sub-themes, and definitions and discuss any areas of discrepancy or needed adjustments. TK then devised the conceptual model through which we present and analyze our findings. Supporting quotes were anonymized, cleaned of fillers and dysfluencies and translated to English if relevant (21). We reviewed this report in light of the consolidated criteria for reporting qualitative research (COREQ) (22).

## Results

**FIGURE 1:**
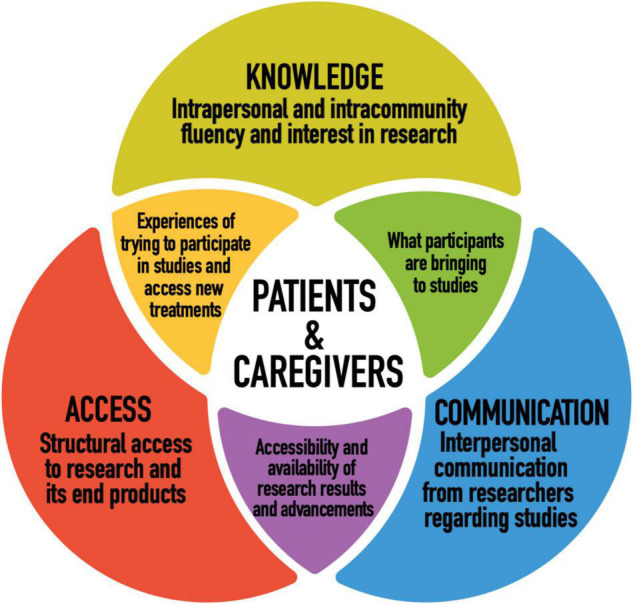
Conceptual model: patient and caregiver perspectives on CF research and personalized medicine.

Figure 1 illustrates our conceptual model. We grouped our findings into three themes that together provide a better understanding of patient and caregiver perspectives on cystic fibrosis research and personalized medicine. Our conceptual model was adapted for our research from systems and ecological models in health communication literature (23–25). Our goal was to best represent what participants, who live directly with or alongside this disease, and who contribute to research through their participation in studies, are bringing to the table in terms of their relationship to and priorities for research. In order to do so, we wanted a model that considers not only what researchers are doing and how they can improve in ethical research design and implementation, but participant agency and how research plays out practically in participant’s lives, beyond the setting of the research institution. We consider all three dimensions of the conceptual model to raise important questions for researchers and policy makers to consider.

### Intrapersonal and Intracommunity Knowledge

#### Background in Personalized Medicine

Most participants, especially within the adult patient subgroup, were relatively well informed about CFTR modulators, including their genetic aspects: *“I have been very excited to see the new developments that are happening, particularly with treating CF on a more cellular level, rather than just treating the major symptoms!”* (P6). They viewed CFTR modulators as heralding a shift in CF care and prognosis: “*I will say that personalized medicine is not only for CF, but just in general is the way of the future. And so, I’m quite hopeful because the Trikafta announcement is the first legit game changer, but it’s the first of many, right? Things will only get better”* (C13). Similarly, they anticipated new modulators will be developed that apply to more and more CFTR genotypes:

*I’ve heard that there’s a lot of new medications, that they’re not just stopping at Trikafta and being like, “OK, well, we’re done now.” It’s good to know that there’s still ongoing research, especially because Trikafta is great for 90% or 95% of us, but there’s still a significant proportion of people who are waiting for their modulator* (P11).

Participants largely felt positively about genetic tests that could assess which medication would be effective, if any - *“It would save us all so much heartache”* (C5)- and voiced hope for personalized medicine to eventually reduce the number of medications they take as well as the side effects they experience. However, opinions were more divided on whether genetic tests should preclude access to existing modulators for people who did not qualify:

*It can be a factor in making the decision which medication to go with or which treatment. But it’s still one factor, there’s adherence to it, there’s other lifestyle factors, there’s medication interactions and then there would be other potential biological interactions that we don’t know about and won’t know about. So, it doesn’t all come down to, it doesn’t all fall on what your DNA says; that would certainly be helpful, but it’s not the whole [picture]* (P18).

Throughout the focus groups there were some misconceptions and misinformation regarding stem cell research, most commonly confusing the use of pluripotent and adult stem cells in research with stem cell therapy or with the controversial use of embryonic stem cells. While responses evinced room for more patient education regarding stem cell research, ultimately most participants had a solid baseline of knowledge in personalized medicine for CF and kept track of emerging medical technologies, citing their development as a source of hope and excitement.

#### Interests in Cystic Fibrosis Research

Patients and their caregivers shared a familiarity with and enthusiasm for CF research. Of those who participated in our focus groups, many patients had participated in research projects throughout their lives, often via their relationships with their local CF clinics. They articulated their motivation to participate in research as a means to help medical science advance for the CF community. In other words, to help improve quality of life and prolong lifespan. As one patient expressed: *“I’m of the mindset that if there’s anything I can do to help anyone in our generation or next, I’m willing to put myself out there and help out as much as possible”* (P7). A significant number of participants were also involved in CF advocacy efforts and viewed research participation as an extension of this.

Participants also largely expressed trust in the ethical conduct of medical CF research teams in Canada.

*Whenever I’ve been asked to participate in research, I’ve always accepted, especially since the standards of medical research in Canada are very high, with the ethics committees, supervision for research work, so that, you know, I’m basically very confident about the process itself* (P21).

Trust in the reliability of research teams to act in their best interest extended to the emergence of personalized medicine. This is not to say that ethical questions and considerations were not important to participants -for example, some had mixed feelings about pharmaceutical companies: *“I feel like we are giving them a broad empty blank check”* (P15). Rather, they felt that existing research protections to be solid enough that the focus can be on moving research forward: *“To me the consequences and the end stage nature of this disease, we know where this leads, to me that overcomes any security [concerns], even potentially some minor ethical issues that maybe otherwise I would be more sensitive to”* (C17). This priority was evident in the way participants typically responded to questions about privacy and future uses: either as a non-issue or in illustrative ways that drove home their priorities to their researcher audience.

I1: *I’m going to ask you again, are there any hesitations or*-
C7: *No [laughs].*
I1: -*are you still, you know. even if it’s identifiable? [*…*] to a certain extent it’s still genetic information and the fact that you have a rare disease that can one day be- we can still pinpoint that that sample came from that child; any hesitations there-*
C7: *No.*
C8: *If it’s not malicious. Like if it’s in the name of research I’m okay with that.*
I2: *The reason we ask that is because there have been ethical debates in the scientific community about that and it’s interesting to have the point of view of those most concerned.*
C7: *Yeah, I understand what you were getting at but at the end of the day if it’s going to help, I don’t care.*

These points are important for researchers because they show a slight disjuncture between research ethics concerns and the primary concerns of the CF community we were interviewing regarding research; we were having two slightly different conversations. As we brought forward research ethics questions, they redirected to and wanted us to understand their interest in CF research advancement as it relates to their lived experience. Within participants’ accounts there was strong awareness of both CF as a chronic progressive and ultimately fatal condition and of the role research has played in improving the health and longevity of patients with CF over the past century: *“I mean the grim reality of CF is that it’s a fatal disease and research is going to take that word out of our vocabulary – I mean that’s the ‘F-word’ in our house [fatal]”* (C16). Interest in research for participants was thus inextricably entangled in the lived experience of CF, including both personal and intracommunity loss.

*When you have a disease like CF and you see your future flashing by- you see the [*…*] other CF [patients] around, who eventually fall to the fight, and then you see time flashing by-, everything you’re able to do yourself to try to make things better*… *You know, if they would have told me, “We’re going to take your arm off, then it’s going to help the research,” I would have said yes* (P21).

The degree to which the CF population is researched- *“they’re always doing something or other”* (P11)- and their orientation toward research as a vehicle for advancements in the field of CF care has implications for ethical research design and implementation that we will return to in the discussion.

### Interpersonal Communication From Researchers

#### Informed Consent

While the majority of patients with CF and caregivers expressed satisfaction with the way research teams handled the initial stages of their participation, a significant thread throughout the focus groups involved insufficient communication regarding informed consent. While miscommunication most often appeared to result from a combination of researcher and participant factors, in order to obtain informed consent, it is essential that participants fully understand the purpose, nature, or very fact of participation.

One example which indicated that communication was unclear or missing was that during the focus groups, several participants brought up that they had participated in clinical trials involving drugs and did not know afterward whether or not they had taken the placebo. It was not clear for them whether the topic had been covered during the consent process or if they should even have had access to this information. Another example was that some participants referenced times where they only had vague understandings of studies they were contributing to and even whether they were participating. Part of the reason some patients and caregivers had confusion or outstanding questions about their research participation relates back to the volume of studies in which they take part as well as overlap with the physical spaces of their medical care.

C8: *It kind of becomes a routine because yeah- like you see so many people during the day and then-*
C7: *Then half the time you don’t even know that you’re in the study.*
C8: [Laughs] *And we sign stuff-*
C7: *Yeah you sign stuff and you don’t even realize that you’re in the study, it’s like ‘okay sure I’m doing a study now.’*

In terms of participant factors, consent forms were described as long and “a lot of jargon” and this, along with participant interest in research advancement, sometimes impacted the way they interfaced with consent processes: “*It’s trying to beat this thing*… *When you go to clinic they hand you a stack of paper, like the research person comes in and says, you know, “Do you consent to us keeping this for CF Canada?” “Sure, I’ll sign that one*” (P13). Other participants wished for more time and space to digest information and ask questions: *“I would have asked more questions. And I couldn’t because everything was done very quickly*…*I would have liked a heads-up coming into clinic that there was research going on and I would have had the time to think about questions”* (P4). It is important to note, in the context of these accounts of suboptimal consent, that patients and caregivers tended to have limits about which studies they would participate in, such as avoiding ones they considered more invasive or that were not directly tied to CF. Overall, thorough informed consent processes from researchers was viewed as symbolic to respect within the research relationship.

*I participated recently in a “trial” (don’t know if it qualifies as research) of a new machine to determine my PFTs. Unfortunately, this experience was unpleasant because I wasn’t explained what was going on and given instructions or an idea of the tests I was doing. The person was unable to explain to me exactly what was happening, and it took several questions before understanding what was going on. This got me extremely frustrated as I was willing to help but felt mistreated and like another “data” set. This was the most recent experience I had with research; however, generally speaking, I have had good experiences with research. For me it all comes down to the information I’m getting (what are we doing, what is it for, what is expected of me and what will happen next)* (P5).

#### Follow Up After Studies

Once the participation phase of research was over, it was rare that participants were aware of the results of the studies in which they took part: “*Do we ever receive the results of those studies? Basically never. We aren’t really included in the follow-up of what the outcomes of the study were”* (C4). There seem to be two main reasons for this. First, there was a lack of follow-up review by research teams with their participants to keep them informed of the progress of the study.

P17: *I’ve never received any follow up that I can think of.*
P16: *I agree with that.*
P17: *You know when you check that box and you say I want to know what the results are.*
P18: *Yeah, me neither. I was just going to add that same here and we’ve come across studies that are, you know [that] we must have been in. So. I too wish there was a little more follow up.*

Second, participants did not have the reflex or did not know, when and how to ask for results: *“For any study I’ve ever been a part of, I’ve never, ever heard of it”* (P11).

There was a general desire on the part of patients and caregivers to have more follow-up from research teams such as a lay summary of findings or notification of associated publications: *“[…] some sort of follow-up would be nice, I guess. I like knowing things. So, even if the study didn’t work out or if it found out the drug was awful or something, I would still be curious to know worst case scenarios just because I find that stuff interesting”* (P15). Many participants reported feeling a sense of pride or satisfaction knowing that their participation may have led to new discoveries. Further, patients form a community where sharing information about updates in ongoing research is important: “[…] *it is very much a community that deals with – has to work against the fact that it’s smaller, in the grander scope of diseases or trials, as well”* (C11). Finally, the extent to which many patients with CF participate in studies can create a sense of reciprocity with research teams. Studies in which participants invest significant time and energy, in particular, become a part of their lives, reinforcing a personal interest in the results, not necessarily to know if there was some new important discovery but mainly to have a follow up and conclusion.

I2: *Would you want to know even if they found nothing?*
C7: *Yes.*
C8: *Yeah.*
C7: *Because [this] study took a lot of time and if they found nothing, I would be mad, like that was a lot of time out of my day for a whole year.*
I2: *Because sometimes the results, you know they’re, sometimes they’re very small.*
C7: *That’s fine. I would want to know because it was every day for a full year twice a day to do the study. That’s a lot of commitment* [laughs]. *I would want to know what they found even if it’s nothing. It was like “well you know there was that”. Yeah I would – yeah because we never find out after, like once it’s done it’s like “well okay, cool”* [laughs].

Some participants allowed that if they had contacted the research team, they would have access to updates. However, they seemed to want the communication of results to be initiated by research teams.

*I’ve never received anything. But, like [p10] said, maybe I never followed-up, so maybe that was on me. If I did follow-up, I would have got a response very quickly. But did it come to me without asking, no* (P9).

Patient and caregiver interest in study results tie back into their interests in research in several important ways. The considerable participation in research of many patients with CF- “*sometimes there’s been a feeling that you are a research study, like your life is a research study*” (C2)- and the size of the CF community refracted onto their investment in receiving study results. It is not only about knowing results but about sharing that information among the CF community and having the research team follow up with their end of research relationship.

### Access to Research and Its End Products

#### Geographical Inequities

Participants brought up inequities and barriers to research participation based on where they live within Canada. They noted that, in Canada, healthcare is administered on a provincial level and some jurisdictions have more resources and better infrastructure than others: *”The problem is that I have a lack of confidence because the health care system in some provinces has more money than others. So, there are some people that- seriously, I really have a lack of confidence that we’re not getting the right care”* (C18). Participants stated further that the facilities with the most cutting-edge research are located in the country’s largest urban centers. Those who live more rurally or remotely described relatively limited local medical services and longer wait times for advancements in healthcare as well as long commutes to the nearest CF clinic, experts and/or hospital: *“I live about 5 hours from the nearest adult CF clinic, so getting there and back every 3 months requires a chunk of time, and then if I need to be hospitalized (which is usually twice a year for 2 weeks each time), then I spend the whole time away from family and friends”* (P6). While they would make the commute to access critical medical care, it was sometimes prohibitive in terms of time, energy, and expenses to make such trips for research:

*I have concerns sometimes in clinical trials about equality of access. I know that there’s a lot of clinical trials that I would have liked to have been a part of, but that weren’t available in my city, right? [They] were only through bigger centers like in Toronto or Vancouver or whatever. When I lived in [—] which is further north in Ontario, they wanted me to come down for a study in Toronto and they were like just make a trip out of it. It’s like are you kidding me? That’s a four-hour drive and hotels* (P16).

Some individuals did end up traveling inter-provincially to access research opportunities:

*We travel to SickKids. They wouldn’t be our closest clinic, but we travel to SickKids specifically because research opportunities are really, really important to me. Obviously, gene modulators are like the holy grail of research studies, but just in general I think with rare diseases research studies are just really important and I very much just want [my child] to have the very best opportunities* (C17).

However, even participants who were able to travel to major research centers were sometimes excluded precisely because they did not live locally: *“We weren’t always included in the research because we were too far away”* (C18). Increasing access to research opportunities at the provincial level was thus a part of the advocacy work of some interviewees.

#### Bureaucratic Factors

Patients and caregivers also expressed frustration with several bureaucratic factors that impacted their access to the end products of research. One was the pace of research. Again, this was inseparable from the lived experience of CF: *“The only thing about research for me is I feel there is a lot of red tape, right? Things take years and years and years, and, for some of us, we don’t have years and years and years”* (P9). They shared several specific criticisms regarding the research to market access pipeline in Canada. First, some felt that ethics review processes could be simplified and made more efficient: “*To me it’s like, knock yourself out, my God you’re going through ethics committees and all these different things and all the hoops. I wish you’d just spend the time getting the research started, or getting it done”* (P22). At the federal level, CFTR modulators, Trikafta in particular, were a sore point for many participants, compounding existing frustrations with the current system of drug approval in Canada:

*If we’re talking about Trikafta or most of the modulators, I find it extremely frustrating to see how things are moving much faster in the United States and Europe than here in terms of access. We have finally reached Health Canada, which is studying the issue, but we are far from having it in our pockets. So, it’s slow. And it’s hard to understand why, because we know that it’s approved elsewhere, so it’s not a question of its effectiveness. I don’t have the impression that we are a priority for the government, and that it is making every effort to ensure that we have access to this drug, which has been shown to change lives* (P19).

Participants further brought up Canada’s Special Access Program, which allows practitioners to request compassionate or emergency access to drugs not yet approved for sale for patients whose illness has become life-threatening (26). While patients or caregivers of patients who had qualified shared accounts of substantial improvement in health and quality of life, they understood their situation as exceptional. In general, stratifying access by severity was not popular among participants and some made contrasts to cancer treatment:

*We would never say to a cancer patient, “We’re going to wait until you’re at stage 4 before we allow you to have the drug”*… *It doesn’t make sense to let our lungs get as bad as they can get, when we could be slowing down the progression of the disease and adding decades to a life. So, what are the reasonable costs for that? It doesn’t make sense* (P19).

Finally, even for medications that have been approved for sale in Canada, patients and caregivers described long arduous processes advocating and sending documentation between their insurance brokers, medical centers, and drug companies in order to gain access to new treatments: *“If I wasn’t vigilant about it on a daily basis, it would not have happened”* (C5). Overall, participants argued that the bench to bedside timeline for crucial and lifesaving treatments evinces problems in research translation on a systemic level:

*There needs to be a mutually beneficial system for all involved: drug manufacturers, insurance companies and government health care systems. The current system of drug approval and price setting obviously does not work, especially for the more targeted treatments that are coming down the pipeline* (P2).

#### Accessibility of Information

Living with CF gives patients and their caregivers expertise in understanding their condition, but this does not necessarily translate into the same level of language that medical professionals use in reporting their findings.

*I would love to know*…*whether there are differences that have been proven to occur depending on the mutation that one has; if other modulators in the pipeline target all types of mutations and other stuff like this. I get discouraged when confronted with medical papers on this because I do feel like I lack knowledge to fully understand, but at the same time, I feel like newspaper articles always say the same thing and don’t go as far as I would like* (P5).

In order to learn more about their condition, patients and caregivers often turn to written publications or information online, most often only available in English and aimed at the scientific community: *“I find that the information that is available is very, very scientific and not made for the general population. It seems to be aimed at people who are already familiar with the subject”* (P19). The problem that emerged from the focus group participant responses was thus not so much the amount of information that exists but its accessibility. There is a double challenge for patients with CF and their families of both finding relevant information and being able to understand it.

*I always wished there was one website that had everything all in one. That would probably be impossible but -that every single researcher worldwide just put everything onto it. It’s like a data dump where you could easily access whatever you needed instead of trying to figure out just where to find anything. Because it’s so impossible to find anything, I find* (C8).

Difficulty finding plain language scientific information about CF research can easily discourage patients and their families from trying to access important information: “*The motivation to search it out and then read through the scientific jargon is just really not there”* (P7). Further, the fragmentation of information through so many different sources (doctors, researchers, the internet, publications) can also lead to differences in the very nature of the information that is given. This can result in misinformation and delay or even prevent the ability of patients and their families to utilize valuable information coming down the pipeline. At the same time participants acknowledged that the specialized and diffuse nature of research presents limits on lay dissemination by researchers: “*I don’t think it’s purposeful to hide it. I think it’s just that there’s not really an accessible way for the general population to be aware of everything that’s going on in research”* (C2). Notably, patient advocacy groups (such as CF Canada and groups on social media) were named as very important in regard to the dissemination of accessible and timely CF information.

#### Financial Inaccessibility

Financial barriers to the end products of research were the most prolific theme under access, particularly regarding CFTR modulators. Respondents described how CF brings added life expenses and limits the ability of many patients and caregivers to work. Individuals with CF are disproportionately in low-income households. They pointed out that the initial costs of these drugs are hugely inaccessible and therefore their access will be dependent on provincial coverage, or, failing that, having private insurance. One participant quipped that in order to have the kind of income necessary to afford Trikafta; *“I would have had to create Trikafta”* (P20). Another person lamented the two-tier system between: *“those who can pay for longer lives and those who cannot”* (P1). CFTR modulators run the risk of exacerbating existing systemic health disparities, not only because of their high price tag, but because they represent the closest thing to a cure:

*The first time I felt fear about my condition was when the Kalydeco announcement came out. It’s a different fear. When you know the cure is there and available, but it’s not accessible to you yet. It’s like I’m in the ocean, we are all together as a group, there are sharks around, and there are people getting eaten as we go along. It’s not the same fear and the same frustration when it’s, “Ah, the helicopter is on its way.” You know, you say, “Okay, well, let’s give it time to get there. That’s fine. I’ll wait, that’s understandable.” But if the helicopter is above, and it’s waiting to let the rope down because people aren’t sure how much it’s going to cost, that’s extremely frustrating. Once the helicopter is there, it could already be saving people* (P21).

For some, the participation of patients in research leading to the development of these medications compounded a further sense of injustice: *“I’ve heard from people who participated in the studies for Orkambi, etc., and saw an upward turn in their health, but when the study ended, they didn’t qualify for continuing the medication after the trial. The only way they could continue this medication that had made a big difference in their health was to pay for it, and the cost was too high to absorb*” (P6).

While they had serious financial access concerns about CFTR modulators, participants still wanted research to continue in this area. Most asserted that their development is invaluable: *“if we don’t do research than nobody’s getting on the drug, period”* (P13). They largely believed these new personalized medicines will eventually become broadly accessible, once patents expire and/or their overall cost effectiveness is demonstrated:

*I also believe that if more drugs are discovered and are proven to work, and eventually prevent the costs of being sick - including hospital stays, transplants, other medications, etc., and if the criteria are modified to take all of this into consideration, these drugs will eventually be covered. Since I do believe that we can change things in that direction (or very strongly hope so), I think it is crucial that researchers continue to develop these drugs* (P5)

However, this did not preclude calls for systemic change in the timeline and costs of access to medication. As one caregiver put it: *“Right now, our mission is access to medication”* (C9). Participants identified the problem as being: *“a system or global discussion to solve with regards to the pharmaceutical industry”* (P16) and asserted that *“patients, as stakeholders should be a part of that solution”* (P22).

## Discussion

**FIGURE 2:**
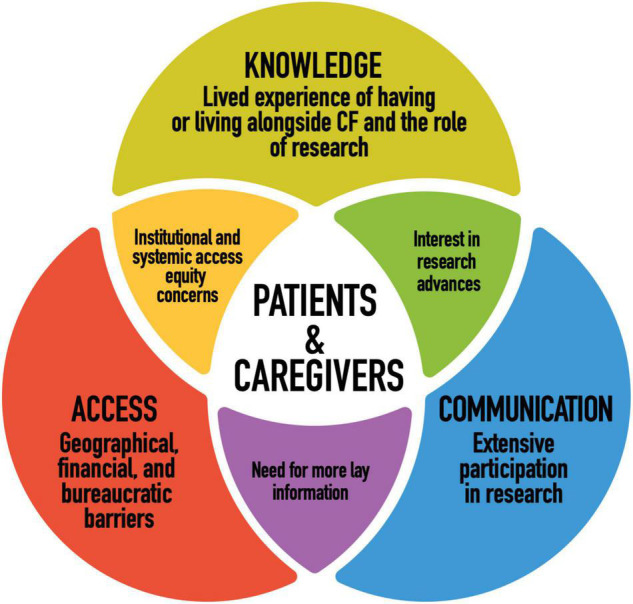
Findings: patient and caregiver perspectives on CF research and personalized medicine.

This empirical study considered the perspectives of patients with CF and their caregivers along three dimensions in order to most fully and dynamically represent their relationship to research. Those were: knowledge (intrapersonal and intracommunity fluency and interest in research), communication (interpersonal communication from researchers regarding studies), and access (structural access to research and its end products). Figure 2 illustrates our main findings.

Participants described a background in and relationship to CF research inextricable from the experience of CF as a lifelong progressive and terminal disease and the goal of advancing medical science. The approach of the CF community to research is thus intertwined with their “fight for life” [p. 356 (27)]. Life not merely as the prevention of death, but life as improved longevity and overall wellbeing. The various ways patients and caregivers articulated their research participation in this study were reminiscent of the findings of Christofides et al. (28) on research participation as a complex helping behavior that simultaneously encompasses prosocial motivations and “a nuanced understanding of the interconnectedness of research and treatment” [p.180 (28)]. Such an interconnected understanding was compounded by the fact that research often takes place in the same settings in which participants receive CF care, that both research participation and care are ongoing aspects of their lifelong medicalization and that progression of their medications and therapies is interwoven with research advancements (27–29). The relationship of participants to CF research thus raises several questions for ethics oversight. On the one hand, some participant accounts venture into therapeutic misconception (i.e., the tendency to misperceive research as treatment). On the other hand, the structural interrelation between research and care for participants as well as their nuanced understandings of the situation echo findings of other studies that question the practicality of binary distinctions between research and care for research ethics including the ideal of a purely altruistic neutral and autonomous research participant (28–32). Overall, the findings of this study highlight the importance of incorporating the lived experience of CF into ethical research design and implementation.

The patients and caregivers we met viewed the emergence of personalized medicine for CF as a major paradigm shift. They were enthusiastic and excited about the emergence of CFTR modulators and emphasized continued research advancement as their primary concern. Focus group discussions about research ethics considerations and personalized medicine brought up two further ethical considerations. The first relates to participants repeated expressed trust in the ethical conduct of Canadian medical researchers. This is a positive finding, especially from those participants who cited decades of research experience. Our findings are also consistent with the results of a recent qualitative research conducted with patients with CF in Europe who show a high degree of trust and a tendency to prioritize research facilitation over other considerations (33). At the same time, participant accounts and illustrative examples de-emphasizing their attention to research ethics (multiple participants only drew the line at the use of research for developing human clones, for example) conversely re-emphasizes the ethical responsibility of researchers to safeguard their rights and wellbeing. This need not be at the expense of the more expedited and accessible ethics process participants asked for but remains an issue of note. Secondly, while participants had a sufficient baseline in the operations regarding CFTR modulators and genomics, their comments evinced a need for more patient/public education on stem cell research and its convergence with genomics, especially if induced pluripotent stem cells (iPSCs) is to be part of new tests and research (34–36).

Participants spoke to how having good communication with researchers is crucial to their relationship with research. The communication during participation in studies may have an impact on how patients feel or experience their disease later. Poor communication with researchers can lead to mistrust and influence future willingness to participate in research. The accounts of patients and caregivers in this study point to the importance of checking with the participants whether the consent form has been understood and whether all questions have been answered. This includes clearer communication regarding the nature of a given study and what will and will not be communicated about the research in which they are enrolled. Participant anecdotes further suggested that more brevity and plainer language on consent forms would be beneficial. Careful and thorough informed consent is especially important in research settings that overlap with clinical care. Participants also wanted to learn about study results (even if nothing significant or new was discovered) and they wanted this to be initiated by the study researchers. Again, this desire comes in part from a more relational understanding of research: participants volunteering their time and bodies via (often familiar) clinics and then expecting to be kept in the loop about published articles and findings. However, they felt this aspect of researcher communication was lacking. Participant requests for greater communication around results has been a theme in previous studies as well as, interestingly, incidents in which participants did not recall that results had been returned, perhaps due to their form of delivery (9, 37). Finally, while it did not come up in focus groups, it is worth noting that since the basis of studies in personalized medicine depends on the genotypic differences between patients, there is the possibility that these studies will uncover important medically actionable individual information for participants and their families. Researchers should have plans in place to deal with these results should they arise (38). Issues of the return of results and incidental findings are complex, however, as they can further blur the lines between research and clinical care.

The access barriers to research and its end products experienced by participants reaffirm those discussed in the literature and offer several concrete points of reflection for researchers (5, 39, 40). First of all, there are inequities of geographical access in Canada. Patients and caregivers asked to have the opportunity to participate in research interprovincially at Canada’s major research centers. The additional time and financial burden for those living more remotely is an argument for travel vouchers or even mobile or multi-site research where possible. Considerations of geographical equity in ethical research design are further important for not compounding the effects of existing disparities in access to CF care and experts in the Canadian context (5). Many participants also argued in favor of more efficient research timelines, including ethics review processes. They believed bureaucratic red tape to be impeding the advancement of research to some degree as well as delaying or obstructing their access to new medications. Participants also had difficulty accessing and understanding much published CF information and wanted researchers to direct more information to lay audiences. Arguably, timely accessible information on CF research is important not only for patient understanding, but for the management of their health. In addition, knowing what is coming down the research pipeline allows patients to better advocate for themselves and the CF community in terms of access to new personalized medicines. The issue of the affordability of the drugs being produced by research also raises several ethical quandaries for researchers, especially given that for many in the CF population, their health condition renders them socioeconomically vulnerable (41). Similar to the lack of effective return of research results, some participants were frustrated with the idea of having helped in the creation of medicines they could not access or afford. It should be noted, however, that the patients and caregivers we spoke with wanted new drugs to continue to be developed regardless because they anticipated they would become accessible eventually. Ultimately, many of the access problems described by participants are systemic and, thus, their resolution would involve the collaboration of multiple stakeholders (policy makers; research and medical institutions; insurance and pharmaceutical companies). Patients with CF and their caregivers explicitly wanted to be part of this process.

### Study Limitations

A qualitative approach for this study was chosen to privilege richness of information. Caution should be taken when extrapolating results from our limited sample: a quantitative approach would have provided greater reliability (but less depth). Similarly, as we collected limited demographic data and, in the interest of confidentiality, did not ask about personal health information, our data did not allow for internal comparative analysis according to individual participant factors such as severity or genotype. Considering that participation in the focus groups was on a self-selected basis, we cannot exclude the possibility that our study population is particularly interested in and knowledgeable about CF research. It is possible that there are greater knowledge gaps about CF and advances in care in the general CF population than our study represented. Further, as we recruited through CF Canada, our participants were, for the most part, engaged in the Canadian CF community in a way that is likely not representative of all people in Canada with CF. While participants were heterogeneous in terms of age, income and education, the relative racial homogeneity of our study population may have precluded the discussion of structural racial inequities in personalized medicine and research even as other structural issues came up (5, 42–44). Finally, as we were asking participants to summarize what was often extensive research experience in one sitting, there may have been recall biases in what was shared.

## Conclusion

The voices of patients with CF and their caregivers are crucial not only in validating or refuting existing concerns in research ethics but in helping to shape the conversation. The findings of this study contribute perspectives, concerns, and priorities of the CF community to the body of work on research about them. Notably also, as a historically and colloquially childhood disease, perspectives of adults living with CF remain particularly scarce (16). There are themes and considerations that emerged in this study that may be applicable to researchers who work in rare and/or progressive diseases more broadly. Of particular interest are the impacts of having a small intercommunicative community with an extensive familiarity with research, overlapping research and medical care settings, and how profoundly the experience of having or living alongside a chronic degenerative disease influenced how participants interfaced with research. Our conceptual model moored research to its implications and circulation not only within but outside the research setting for participants, providing additional layers of context for issues such as informed consent and the return of results. It also raised broader questions regarding what duty researchers have to make research and its end products accessible, not only specific studies and lay dissemination of advancements in the field, but also in terms of advocacy for equitable access to the medicines research produces. Our conceptual model could be applicable to other research ethics projects interested in engaging with research systemically.

Since our focus groups concluded in March 2021, there have been some substantial developments in Canada. In June 2021, Health Canada granted Trikafta market approval. As of November 2021, every province and territory in Canada had committed to fund Trikafta for eligible people on their public drug plans. These developments are significant gains for the CF community in Canada. The focus of CF Canada has shifted to advocating for improved access (as access qualifications and the relative accessibility of reimbursement systems vary by jurisdiction) as well as for private insurers to provide coverage (45). Issues of research ethics and research translation in personalized medicine will continue to be critical as the landscape of CF care shifts in this direction. This includes the need for the development of multi-media educational tools for patients with CF that are readily available and describe current research developments in lay terms. It will also entail new points of consideration for research and research ethics as more people with CF enter later adulthood. Ultimately, better mutual understanding among researchers, policy-makers, and the CF community is necessary to effective, accessible and relevant research advancement.

## Data Availability Statement

The transcripts of focus groups for this study will not be made publicly available. Focus group transcripts cannot be fully anonymized and participants did not provide consent for the sharing of transcripts with parties other than the researchers. The complete codebook will be available upon request. Requests to access the codebook should be directed to TK, theresa.knoppers@mcgill.ca.

## Ethics Statement

This study involving human participants was reviewed and approved by the Research Ethics Board of McGill University’s Faculty of Medicine (IRB Study Number A11-B57-18B). Participants provided both written and audio-recorded verbal informed consent.

## Author Contributions

BMK, MTN, and JH conceived, designed, and obtained the funding for this study. JH obtained ethical approval for the study. MC bottom-lined participant recruitment. JH, MC, and MTN facilitated the focus groups as moderators. JH conducted the qualitative thematic analysis, which was validated by TK and MC. TK created the conceptual model and drafted the manuscript. JH and MC also contributed to writing specific sections of the manuscript. All authors participated in revisions and have read and approved the final draft.

## Conflict of Interest

The authors declare that the research was conducted in the absence of any commercial or financial relationships that could be construed as a potential conflict of interest.

## Publisher’s Note

All claims expressed in this article are solely those of the authors and do not necessarily represent those of their affiliated organizations, or those of the publisher, the editors and the reviewers. Any product that may be evaluated in this article, or claim that may be made by its manufacturer, is not guaranteed or endorsed by the publisher.

## References

[1] Ratjen F Bell SC Rowe SM Goss CH Quittner AL Bush A. Cystic fibrosis. *Nat Rev Dis Primers.* (2015) 1:15010. 10.1038/nrdp.2015.10 27189798 PMC7041544

[2] Cystic Fibrosis Canada. *The Canadian Cystic Fibrosis Registry 2019 Annual Data Report.* (2020). Available online at: https://www.cysticfibrosis.ca/registry/2019AnnualDataReport.pdf (accessed November 30, 2021).

[3] Lopes-Pacheco M. CFTR modulators: the changing face of cystic fibrosis in the era of precision medicine. *Front Pharmacol.* (2020) 10:1662. 10.3389/fphar.2019.01662 32153386 PMC7046560

[4] Bell SC Mall MA Gutierrez H Macek M Madge S Davies JC Future of cystic fibrosis care: a global perspective. *Lancet Respir Med.* (2020) 8:65–124. 10.1016/S2213-2600(19)30337-6 31570318 PMC8862661

[5] Shemie G Nguyen MT Wallenburg J Ratjen F Knoppers BM. The Equitable implementation of cystic fibrosis personalized medicines in Canada. *J Pers Med.* (2021) 11:382–94. 10.3390/jpm11050382 34067090 PMC8151662

[6] Joshi D Ehrhardt A Hong JS Sorscher EJ. Cystic fibrosis precision therapeutics: emerging considerations. *Pediatr Pulmonol.* (2019) 54:S13–7. 10.1002/ppul.24547 31715091 PMC6871648

[7] Saiman L. Improving outcomes of infections in cystic fibrosis in the era of CFTR modulator therapy. *Pediatr Pulmonol.* (2019) 54:S18–26. 10.1002/ppul.24522 31715086

[8] Cirilli N Buzzetti R Costa S Magazzu G. 282 patient priorities for research in cystic fibrosis: the IPaCOR experience. *J Cyst Fibros.* (2014) 13:S120. 10.1016/S1569-1993(14)60417-3

[9] Christofides E Stroud K Tullis DE O’Doherty KC. Improving dissemination of study results: perspectives of individuals with cystic fibrosis. *Res Ethics.* (2019) 15:1–14. 10.1177/1747016119869847

[10] Rowbotham NJ Smith S Leighton PA Rayner OC Gathercole K Elliott ZC The top 10 research priorities in cystic fibrosis developed by a partnership between people with cf and healthcare providers. *Thorax* (2018) 73:388–90. 10.1136/thoraxjnl-2017-210473 28778919 PMC5870449

[11] Budin-Ljøsne I Harris JR. Patient and interest organizations’ views on personalized medicine: a qualitative study. *BMC Med Ethics.* (2016) 17:28. 10.1186/s12910-016-0111-7 27178188 PMC4866041

[12] American Society of Human Genetics (ASHG). *Public Attitudes Toward Genetics & Genomics Research: Literature and Polling Review Report.* (2020). Available online at: https://www.ashg.org/wp-content/uploads/2020/01/2020-Public-Views-Genetics-Literature-Review.pdf (accessed November 30, 2021).

[13] Jones AM Favaro A St Philip E. *This Drug Can Treat 90 Percent of Cystic Fibrosis Patients, But It’s Not Available in Canada. CTV News.* (2020). Available online at: https://www.ctvnews.ca/health/this-drug-can-treat-90-per-cent-of-cystic-fibrosis-patients-but-it-s-not-available-in-canada-1.4991938 (accessed November 30, 2021).

[14] Lensch MW. Public perception of stem cell and genomics research. *Genome Med.* (2011) 3:44. 10.1186/gm260 21745416 PMC3221543

[15] Marcon AR Murdoch B Caulfield T. Fake news portrayals of stem cells and stem cell research. *Regen Med.* (2017) 12:765–75. 10.2217/rme-2017-0060 29115183

[16] Varilek BM Isaacson MJ. The dance of cystic fibrosis: experiences of living with cystic fibrosis as an adult. *J Clin Nurs.* (2020) 29:3553–64. 10.1111/jocn.15397 32608531

[17] Krueger RA Casey MA. *Focus Groups: A Practical Guide for Applied Research.* Thousand Oaks, CA: Sage (2000). p. 215.

[18] Saiman L Siegel JD LiPuma JJ Brown BF Bryson EA Chambers MJ Infection prevention and control guidelines for cystic fibrosis: 2013 update. *Infect Control Hosp Epidemiol.* (2014) 35:S1–67. 10.1086/676882 25025126

[19] Cook K Jack S Siden H Thabane L Browne G. Innovations in research with medically fragile populations: using bulletin board focus groups. *Qual Rep.* (2014) 19:1–12. 10.46743/2160-3715/2014.1000

[20] Williams S Clausen MG Robertson A Peacock S McPherson K. Methodological reflections on the use of asynchronous online focus groups in health research. *Int J Qual Methods.* (2012) 11:368–83. 10.1177/160940691201100405

[21] Riessman CK. *Narrative Methods for the Human Sciences.* Thousand Oaks, CA: Sage (2008). p. 251.

[22] Tong A Sainsbury P Craig J. Consolidated criteria for reporting qualitative research (COREQ): a 32-item checklist for interviews and focus groups. *Int J Quality Health Care.* (2007) 19:349–57. 10.1093/intqhc/mzm042 17872937

[23] Brofenbrenner U. Toward an experimental ecology of human development. *Am Psychol.* (1977) 32:513–31. 10.1037/0003-066X.32.7.513

[24] McLeroy KR Bibeau D Steckler A Glanz K. An ecological perspective on health promotion programs. *Health Educ Q.* (1988) 15:351–77. 10.1177/109019818801500401 3068205

[25] Sallis JF Owen N Fisher EB. Ecological models of health behavior. In: Glanz K Rimer BL Viswanath K editors. *Health Behavior and Health Education: Theory, Research, and Practice.* San Francisco, CA: Jossey-Bass (2008). p. 465–86.

[26] Government of Canada. *Special Access Program.* (2021). Available online at: https://www.canada.ca/en/health-canada/services/drugs-health-products/special-access/drugs/special-access-programme-drugs.html (accessed November 30, 2021).

[27] Jessup M Parkinson C. “All at sea”: the experience of living with cystic fibrosis. *Qual Health Res.* (2010) 20:352–64. 10.1177/1049732309354277 19955225

[28] Christofides E Stroud K Tullis DE O’Doherty KC. The meanings of helping: an analysis of cystic fibrosis patients’ reasons for participating in biomedical research. *J Empir Res Hum Res Ethics.* (2017) 12:180–90. 10.1177/1556264617713098 28593817

[29] Dobson JA Christofides E Solomon M Waters V O’Doherty KC. How do young people with cystic fibrosis conceptualize the distinction between research and treatment? A qualitative interview study. *AJOB Empir Bioeth.* (2015) 6:1–11. 10.1080/23294515.2014.997898

[30] Hallowell N Cooke S Crawford G Lucassen A Parker M Snowdon C. An investigation of patients’ motivations for their participation in genetics-related research. *J Med Ethics.* (2010) 36:37–45. 10.1136/jme.2009.029264 20026692

[31] Kass NE Faden RR Goodman SN Pronovost P Tunis S Beauchamp TL. The research treatment distinction: a problematic approach for determining which activities should have ethical oversight. *Hastings Cent Rep.* (2013) 43:S4–15. 10.1002/hast.133 23315895

[32] Olsen L DePalma L Evans JH. Self-interested and altruistic motivations in volunteering for clinical trials: a more complex relationship. *J Empir Res Hum Res Ethics.* (2020) 15:443–51. 10.1177/1556264620914463 32363984

[33] Lensink MA Boers SN Gulmans VA Jongsma KR Bredenoord AL. Mini-gut feelings: perspectives of people with cystic fibrosis on the ethics and governance of organoid biobanking. *Per Med.* (2021) 18:241–54. 10.2217/pme-2020-0161 33825546

[34] Ahmadi S Bozoky Z Di Paola M Xia S Li C Wong AP Phenotypic profiling of CFTR modulators in patient-derived respiratory epithelia. *NPJ Genom Med.* (2017) 2:1–10. 10.1038/s41525-017-0015-6 28649446 PMC5481189

[35] Chen KG Zhong P Zheng W Beekman JM. Pharmacological analysis of CFTR variants of cystic fibrosis using stem cell-derived organoids. *Drug Discov Today.* (2019) 24:2126–38. 10.1016/j.drudis.2019.05.029 31173911 PMC6856431

[36] Eckford PD McCormack J Munsie L He G Stanojevic S Pereira SL The cf canada-sick kids program in individual cf therapy: a resource for the advancement of personalized medicine in CF. *J Cyst Fibros.* (2019) 18:35–43. 10.1016/j.jcf.2018.03.013 29685812

[37] Richter G Krawczak M Lieb W Wolff L Schreiber S Buyx A. Broad consent for health care–embedded biobanking: understanding and reasons to donate in a large patient sample. *Genet Med.* (2018) 20:76–82. 10.1038/gim.2017.82 28640237

[38] Thorogood A Dalpé G Knoppers BM. Return of individual genomic research results: are laws and policies keeping step? *Eur J Hum Genet.* (2019) 27:535–46. 10.1038/s41431-018-0311-3 30622328 PMC6460582

[39] Balfour-Lynn IM. Personalized medicine in cystic fibrosis is unaffordable. *Paediatr Respir Rev.* (2014) 15:2–5. 10.1016/j.prrv.2014.04.003 24832698

[40] Oates GR Schechter MS. Socioeconomic status and health outcomes: cystic fibrosis as a model. *Expert Rev Respir Med.* (2016) 10:967–77. 10.1080/17476348.2016.1196140 27268142

[41] McGarry ME Williams IIWA McColley SA. The demographics of adverse outcomes in cystic fibrosis. *Pediatr Pulmonol.* (2019) 54:S74–83. 10.1002/ppul.24434 31715087 PMC6857719

[42] Geneviève LD Martani A Shaw D Elger BS Wangmo T. Structural racism in precision medicine: leaving no one behind. *BMC Med Ethics.* (2020) 21:17. 10.1186/s12910-020-0457-8 32075640 PMC7031946

[43] McGarry ME McColley SA. Cystic fibrosis patients of minority race and ethnicity less likely eligible for CFTR modulators based on CFTR genotype. *Pediatr Pulmonol.* (2021) 56:1496–503. 10.1002/ppul.25285 33470563 PMC8137541

[44] Popejoy AB Fullerton SM. Genomics is failing on diversity. *Nature.* (2016) 538:161–4. 10.1038/538161a 27734877 PMC5089703

[45] Cystic Fibrosis Canada. *News.* (2022). Available online at: https://www.cysticfibrosis.ca/news (accessed February 9, 2022).

